# A simplified single transseptal puncture approach using high-density 3D voltage mapping for atrial fibrillation ablation: acute complications and long-term results

**DOI:** 10.3389/fcvm.2023.1309900

**Published:** 2023-11-23

**Authors:** Pedro Silva Cunha, Bárbara Lacerda Teixeira, Sérgio Laranjo, Guilherme Portugal, Bruno Valente, Ana Sofia Delgado, Mariana Pereira, António Condeixa Rocha, Manuel Brás, Madalena Coutinho Cruz, Margarida Paulo, Ana Lousinha, Cátia Guerra, Rui Cruz Ferreira, Mário Martins Oliveira

**Affiliations:** ^1^ Arrhythmology, Pacing and Electrophysiology Unit, Cardiology Service, Santa Marta Hospital, Centro Hospitalar Universitário Lisboa Central, Lisbon, Portugal; ^2^ Clínica Universitária de Cardiologia, Centro Clínico Académico de Lisboa, Lisboa, Portugal; ^3^ Instituto de Fisiologia, Faculdade de Medicina, Universidade de Lisboa, Lisbon, Portugal; ^4^ CCUL@RISE, Faculdade de Medicina, Universidade de Lisboa, Lisbon, Portugal; ^5^ Comprehensive Health Research Center, NOVA Medical School, Faculdade de Ciências Médicas, NMS, FCM, Universidade NOVA de Lisboa, Lisbon, Portugal; ^6^ Departamento de Fisiologia, NOVA Medical School, Faculdade de Ciências Médicas, NMS, FCM, Universidade NOVA de Lisboa, Lisboa, Portugal; ^7^ Biosense Webster, Lisbon, Portugal

**Keywords:** atrial fibrillation, catheter ablation, transseptal puncture, complications, safety

## Abstract

**Background:**

An ablation catheter and a circular mapping catheter requiring a double transeptal puncture (TSP) for left atrial access have been conventionally used for atrial fibrillation (AF) ablation. Recently, different operators have combined a single transseptal puncture technique with 3D high-density mapping catheters for pulmonary vein isolation (PVI).

**Objective:**

This study aims to compare two strategies, single vs. double TSP, regarding the duration of the procedure, radiation time, complication rates, and outcomes.

**Methods:**

Retrospective analysis of a large cohort of consecutive patients that underwent first PVI with radiofrequency energy (RF), using a point-by-point strategy, with a 3D mapping system, either with single or double TSP, according to the operator's choice.

**Results:**

285 patients with a mean age of 59.5 ± 11.6 years (36.5% female, 67.7% paroxysmal AF) underwent a point-by-point catheter ablation with RF between July 2015 and March 2020. The mean CHA2DS2-VASc score was 1.7 ± 1.3. Single TSP was performed in 115 (40.3%) patients and double TSP in 170 (59.6%). The operator's experience (≥5 years of AF ablation procedures) was equally distributed among the two groups. The average procedure time (133 ± 31.7 min vs. 123 ± 35.5 min, for single and double TSP, respectively) did reach a statistical difference between both groups (*p* = 0.008), but there was a substantial advantage regarding fluoroscopy time (13 ± 6.3 min vs. 19 ± 9.1 min, for single and double TSP, respectively; *p* < 0.001). Acute major complications present similar rates in both groups (2.6% vs. 2.3%, *p* = 0.799). At the 2-year follow-up, both groups had a similar sinus rhythm maintenance rate (76.5% vs. 78.8%, *p* = 0.646).

**Conclusion:**

A simplified single-TSP technique using high-density multi-electrode 3D mapping is a safe and highly successful option for AF ablation. This approach yields a substantial reduction in fluoroscopy time, with the potential to avoid acute complications, compared to a conventional double-TSP strategy.

## 1. Introduction

In the past two decades, significant advancements have been made in managing atrial fibrillation (AF) through catheter ablation. Initially, the procedure focused on isolating pulmonary veins (PV) due to their role in AF initiation (1). So, complete PV isolation (PVI) became the foundation for catheter ablation in AF therapy (2) and circumferential ablation around the PV ostia has become a key element in AF ablation. Catheter ablation is now a routine procedure with a high success rate and a low incidence of complications (3–5). Randomised trials have shown its superiority over antiarrhythmic drugs when carefully selecting patients (6–12). As a result, PVI is widely performed as an effective treatment for AF ablation, with current ESC guidelines recommending it as a primary strategy for all AF patients undergoing ablation (13).

Radiofrequency (RF) point-by-point ablation guided by electro-anatomical mapping systems is standard in most electrophysiology laboratories. High-density mapping catheters with more electrodes and smaller distances between them have emerged (14), allowing for more precise and faster mapping and offering detailed insights into arrhythmia mechanisms (15, 16).

Transeptal (TSP) access to the left atrium (LA) remains a challenging step in the ablation procedure (17), with inherent risks and safety concerns, including the major complication of cardiac perforation and cardiac tamponade (18, 19). The original technique for TSP, introduced by Ross (20), is still the standard but demands a high level of operator skill and a steep learning curve.

In many centres, a standard approach for AF ablation involves two sequential TSP punctures (21). Double TSP access allows the insertion of two catheters simultaneously, aiding in real-time visualisation of PV electrograms without needing catheter changes. Another option is a simplified ablation strategy using a single TSP with the multipolar mapping catheter and the ablation catheter through a single sheath (one catheter at a time).

In this study, we assessed the feasibility, safety, and long-term outcomes of a single TSP technique using a high-density mapping catheter. We compared it with the traditional double TSP strategy for AF ablation.

## 2. Methods

### 2.1. Study population

We performed a retrospective analysis of a non-randomized cohort of 285 consecutive patients undergoing PVI procedures (without additional left atrial lesions) with point-by-point radiofrequency to treat paroxysmal or persistent AF. The database was registered at the local Institutional Review Board. The hospital ethics committee approved the study protocol (Ethics Committee approval number 974/2020). All participants provided written consent for data collection, and the analysis was conducted according to the Declaration of Helsinki guidelines.

We studied data from all patients over 18 years with AF diagnosis, refractory to at least one anti-arrhythmic drug agent, undergoing catheter ablation as a first procedure from July 2015 to March 2020. Patients with a left atrial flutter, undergoing AF ablation with a single-shot technique or repeated ablation (re-do) were excluded from the current study. AF ablation was performed using a 3-dimensional electroanatomic mapping system (CARTO, Biosense Webster, Inc., Diamond Bar, CA and EnSite NavX/Velocity, Abbott Laboratories, Abbott Park, IL).

Single TSP was performed in 115 patients, and double TSP in 170 cases. Information was collected regarding demographics, anthropometric data, baseline bleeding risk, anti-arrhythmic drugs, LA dimensions, left ventricular ejection fraction (LVEF), and structural heart disease. Regarding the comparison between both techniques, we considered the complication rates as a primary safety endpoint and, as a secondary efficacy endpoint, the freedom from any documented sustained atrial tachyarrhythmia (AF/AT) episode during a 2-year follow-up.

### 2.2. Ablation procedure

Every patient underwent a routine preprocedural transthoracic echocardiogram to evaluate LVEF and LA dimensions and to screen for structural heart disease. Computed tomography (with LA segmentation) was performed to assess LA anatomy and exclude the presence of intracardiac thrombi. Additionally, if the mentioned imaging study was obtained >48 h before the procedure, transesophageal echocardiography was performed on the day of the ablation to exclude thrombi. All patients underwent ablation with continued oral anticoagulation (at least four weeks before ablation), using warfarin to maintain an international normalised ratio between 2.0 and 3.0. If the patient was under direct oral anticoagulants (DOAC), the last dose was omitted on the day before the ablation.

All the procedures were carried out in conscious sedation and analgesia, using propofol infusion and fentanyl or under general anaesthesia. A deflectable decapolar catheter was positioned through the right femoral vein into the coronary sinus (CS) to guide the TSP, record, and pace the LA. Continuous monitoring of oxygen saturation and ECG were maintained throughout the ablation. Three experienced operators (≥5 years of AF ablation procedures) performed all catheter ablation interventions, and the number of procedures carried out by each of the operators was similarly distributed in both the single and double TSP groups (operator 1 = 102, operator 2 = 87 and operator 3 = 96 total procedures respectively).

Power settings were at the individual operator’s discretion within the 25–40 W range, depending on the LA segment (25–30 W posterior wall; 30–40 W anterior wall). Ablation Index (Biosense Webster, Inc, Diamond Bar, CA) (22) targets guided each lesion: 480 at the roof and anterior walls and 380 at the posterior and inferior walls and LSI (23, 24) (Abbott, Abbott Park, IL) with target LSI values at 4.5 and 4.0 for anterior and posterior walls, respectively.

#### 2.2.1. Double transseptal—double sheath technique

**Figure 1:**
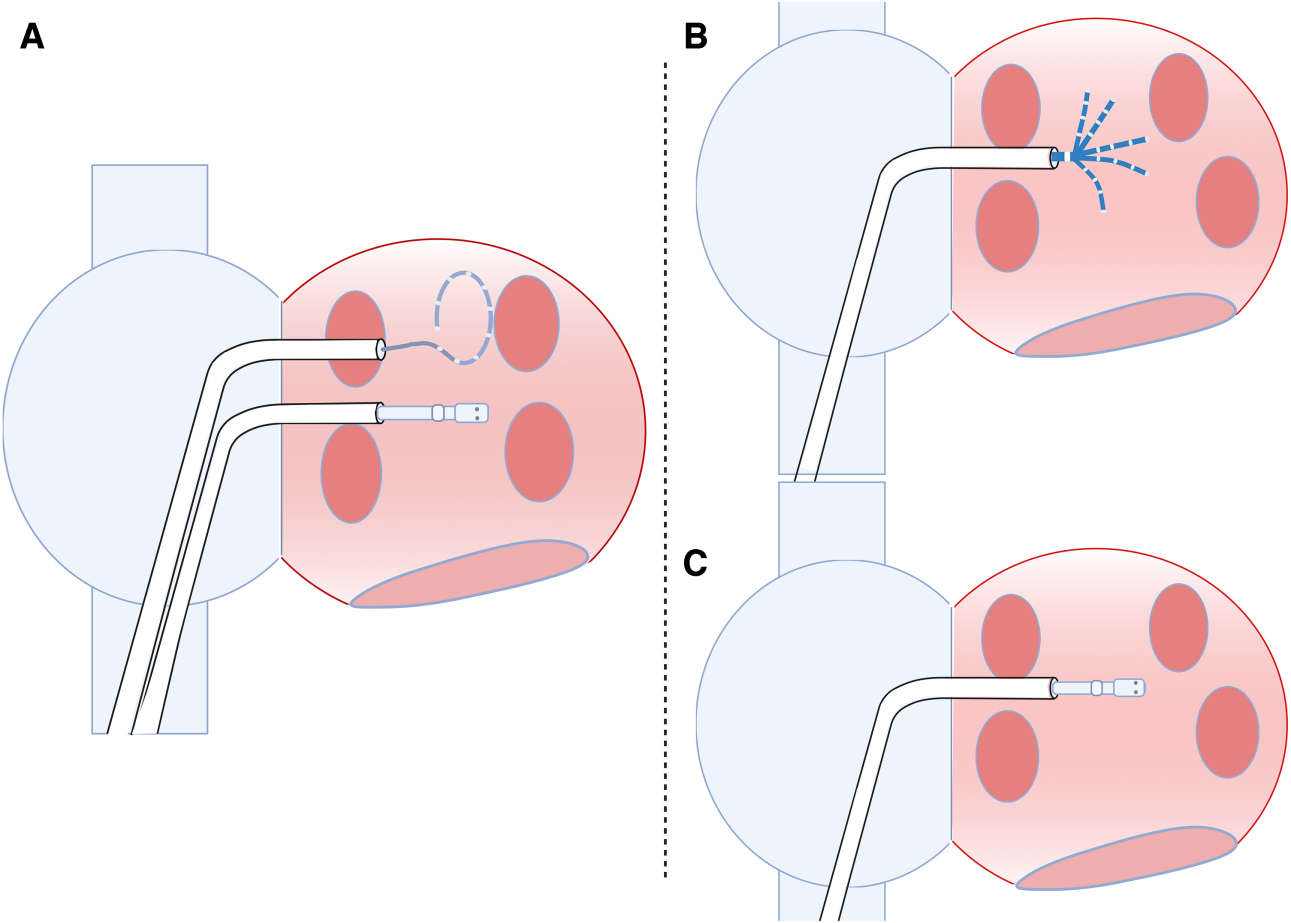
Types of punctures for LA access (double and single puncture). (**A**) Two punctures in the inter-atrial septum for left atrium access. (**B**) Single transeptal puncture with the mapping catheter used to create LA geometry and high-density voltage mapping. After completing the geometry and voltage map, the mapping catheter is removed, and the ablation catheter is introduced (**C**) by the same sheath.

Catheter access was performed using 7, 8 and 8.5 French (F) sheaths inserted in the right femoral vein. During TSP, a decapolar diagnostic catheter was placed in the CS for mapping reference and orientation. TSP was done using a needle system (89 cm, BRK transseptal needle, St. Jude Medical, St. Paul, MN, USA) and a non-deflectable 8 and 8.5 French sheaths SL-1 (Fast-CathTM Transseptal Guiding Introducer SwartzTM SL, Abbott, St. Paul, MN, USA) with fluoroscopic guidance. The 8 F sheath was introduced, and the needle was placed into the superior vena cava and then pulled down. Two movements are detected: the entrance into the right atrium and the fossa oval (FO). After FO identification, the puncture was performed (in the left anterior oblique view). After the first puncture, the same needle system and an 8.5 F introducer (8.5 F, Abbott, St. Paul, MN, USA) were used for the second TSP, performed the same as the described first transseptal puncture. After having both sheaths in the left atrium, a circular mapping catheter and an ablation catheter were inserted through the transseptal sheaths (Figure 1A).

Both sheaths were continuously irrigated with saline during the procedure. Heparin was administered intravenously as a bolus (100 U/kg of standard heparin) immediately after TSP, followed by an additional bolus to maintain an activated clotting time of ≥300 s. Three-dimensional mapping of the LA was performed with either the EnSite NavX/Velocity (Abbott Laboratories, Abbott Park, IL) or CARTO (Biosense Webster, Irvine, California) system. The mapping catheter used was Inquiry^TM^ steerable (Abbott) or the Lasso® (Biosense Webster). Radiofrequency applications were performed using an open-irrigated-tip catheter (TactiCath Ablation Catheter®, Sensor Enabled or ThermoCool SmartTouch® _SurroundFlow, Biosense Webster) with point-by-point-lesions. To verify PVI, stimulation with 10 V outputs was delivered through all pairs of electrodes of the circular mapping catheter positioned beyond the ablation line (exit block). Also, pacing from close to the ablation line was performed to confirm electrical isolation (entrance block) (25). Additional applications were delivered if conduction gaps were identified. A successful procedure was defined by the absence of PVI-LA conduction at all pacing sites.

#### 2.2.2. Single transseptal—single sheath technique

Catheter access was performed by 7 and 8.5 French sheaths inserted in the right femoral vein. In addition, a decapolar diagnostic catheter was placed in the CS for mapping reference and orientation during TSP. A single TSP was done by fluoroscopic guidance using a needle system (98 cm, BRK transseptal needle, Abbott, St. Paul, MN, USA) and an Agilis NxT steerable introducer (8.5 F, Abbott, St. Paul, MN, USA), medium (22.4 mm) curl Dual-Reach™ Bi-directional.

Heparin was administered intravenously as described above. The Agilis sheath was continuously irrigated with heparinised saline during the whole procedure. The high-density mapping catheter (CARTO®PENTARAY®, Biosense Webster, Inc., Baldwin Park, CA, USA) was introduced into the LA through the Agilis sheath, and the 3D mapping system (CARTO®, Biosense Webster, USA) and used to create the LA geometry and a high-density voltage mapping. Mapping was done during sinus rhythm or distal CS pacing with the following settings: filtering cycle length: 550–650 ms, LAT stability: 5 ms, position stability: 5 mm, density: 1 mm, voltage scale: <0.2 mV.

**Figure 2:**
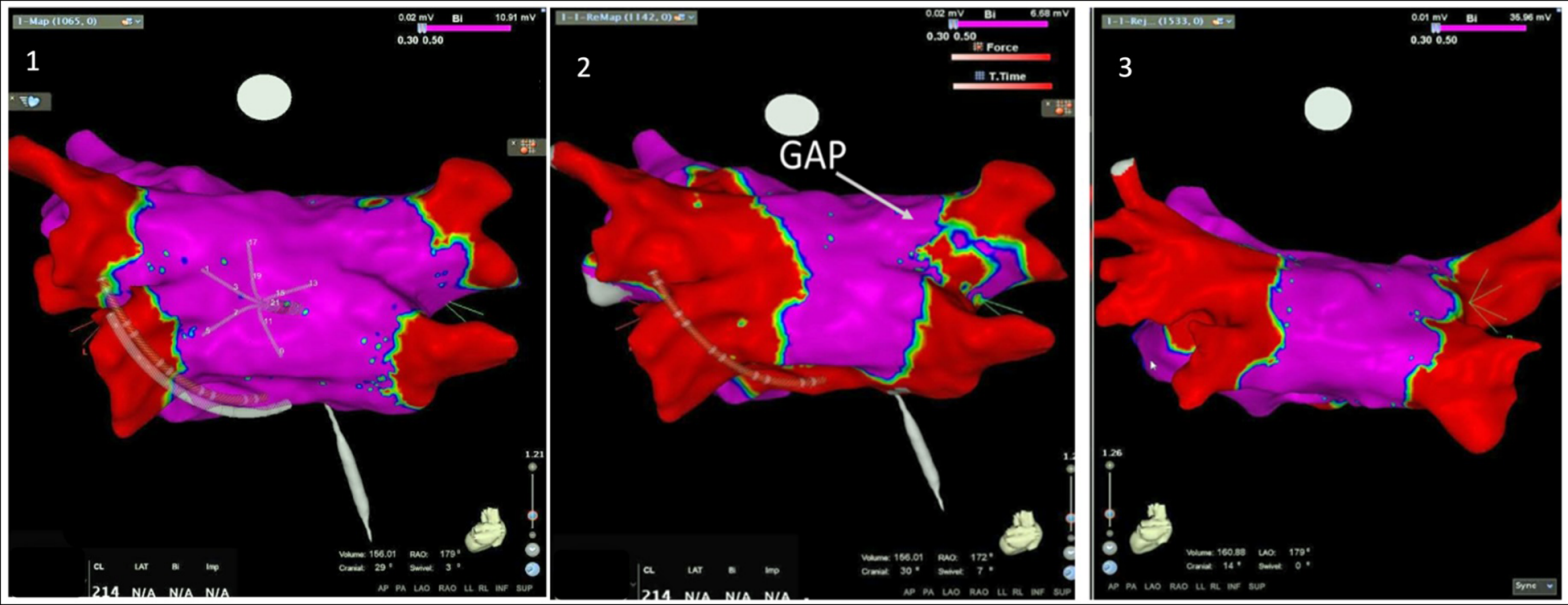
Electroanatomic mapping using a high-density mapping catheter. Posterior-anterior view of left atrial voltage map with CARTO 3-D mapping system. Panel 1: basal map collected with a high-density catheter (PentArray). Panel 2: second map, after the first pass ablation, with identification of a gap in the ablation line in the posterior aspect of the right superior pulmonary vein. Panel 3: map after the second pass of radiofrequency applications, showing complete isolation of both pairs of veins.

After completing the geometry and voltage map, the mapping catheter was removed, and the ablation catheter was introduced by the same sheath (Figures 1B,C). RF ablation was performed using an open-irrigated SmartTouch catheter (Biosense Webster, Inc., Baldwin Park, CA, USA, 3.5 mm open-irrigated tip), by a point-by-point approach, with the catheter in a stable position and a numeric contact force value of ≥10 g. Maximum delivered RF energy did not exceed 40 W, provided by a 500 kHz ablation unit (Stockert EP shuttle, Biosense Webster, Inc., Baldwin Park, CA, USA). Next, remapping was performed to analyse signals, possible gaps and low-voltage areas. If so, the ablation of gaps was followed by remapping to confirm homogeneous low-voltage and PVI (Figure 2). A bi-directional block and low voltage homogeneity of PV and antrum proved PVI.

### 2.3. Post-ablation follow-up

After the ablation procedure, patients were discharged on AAD at the operator’s discretion, together with oral anticoagulation. Patients were observed for routine follow-up in the outpatient clinic 1–3 months after the procedure and every six months (or earlier if symptoms) during the first two years post-ablation. At each visit, a standard 12-lead ECG was obtained. After the blanking period, patients were followed up with a 24 h Holter at each outpatient visit. AAD was continued for six months after CA and was withdrawn—except for beta-blockers—if the patients were free from arrhythmia-related symptoms. Oral anticoagulation was re-evaluated in the third month, and the decision to continue was based on the CHA2DS2-VASc score. In addition, clinical events occurring during the follow-up were evaluated. Atrial arrhythmia recurrence was defined as any documented episode of AF or atrial tachycardia (AF/AT) sustained for >30 s However, symptomatic and clinically typical sustained episodes were considered recurrences even without documentation.

## 3. Statistical analysis

Descriptive statistics summarised patients’ characteristics, procedural data, safety, and follow-up. Continuous variables were expressed as mean ± SD, and categorical data as frequencies and percentages. Intergroup analysis was made using an unpaired *t*-test, and intragroup analysis using the Chi-square test. The Chi-square test was used for the comparison of nominal variables. The student *t*-test and one-way ANOVA were used to compare continuous variables. Comparisons were made using non-missing data only. The Kaplan–Meier model was used to determine cumulative estimates of AF/AT recurrence following the 90-day blanking period through a 2-year follow-up, with between-group comparisons of cumulative event rates calculated using the log-rank test. All tests were two-sided, and a statistical significance was considered when *P* < 0.05. Analyses were performed using Stata, version 14.1 (StataCorp, College Station, TX).

## 4. Results

### 4.1. Baseline characteristics

We enrolled 285 consecutive patients who underwent catheter ablation utilising point-by-point radiofrequency (RF) energy at our institution. In this cohort study, the average age of the patients was 59.5 ± 11.6 years, and 36.5% were female (as indicated in Table 1). Among the patients, 193 (67.7%) had paroxysmal atrial fibrillation (AF); the mean CHA2DS2-VASc score was 1.7 ± 1.3 points. At the time of ablation, all patients were receiving oral anticoagulation therapy. Single transeptal puncture (TSP) was performed in 115 (40.3%) patients, while double TSP was performed in 170 (59.6%) patients. The double TSP group had a higher prevalence of paroxysmal AF. There were no statistically significant differences between the groups regarding age, body mass index, or gender. This procedure represented the initial ablation for all patients.

**Table 1:** Baseline sample characteristics.

Variable	Overall sample (*n* = 285)	Single puncture (*n* = 115)	Double puncture (*n* = 170)	*p*-value
Age (years)—mean ± SD	59.5 ± 11.6	60.8 ± 10.6	58.6 ± 12.3	0.111
Female, *n* (%)	104 (36.5)	41 (35.7)	63 (37.1)	0.809
BMI (kg/m^2^)–median (IQR)	27.9 (5.6)	28 (6)	27.8 (5)	0.946
paroxysmal AF, *n* (%)	193 (67.7)	65 (56.5)	128 (75.3)	<0.001
persistent AF, *n* (%)	92 (32.3)	50 (43.5)	42 (24.7)	<0.001
CHA_2_DS_2_-VASC—mean ± SD	1.7 ± 1.3	1.9 ± 1.3	1.5 ± 1.2	0.035

BMI, body mass index; AF, atrial fibrillation.

Regarding comorbidities (as displayed in Table 2), the groups did not exhibit significant differences concerning conditions such as hypertension, hyperlipidemia, diabetes mellitus, obstructive sleep apnea, chronic obstructive pulmonary disease (COPD), thyroid disease, cancer, heart failure, coronary disease, vascular disease (including stroke, peripheral artery disease, and aortic plaques), and hypertrophic cardiomyopathy. Furthermore, the left ventricular ejection fraction did not display statistically significant differences between the groups. The only variations between the groups were observed in the occurrences of hyperlipidemia and a history of previous stroke or transient ischemic attack, which are not expected to impact the interpretation of the results significantly. On the other hand, the CHADS-VASC score was higher in the single puncture group. As assessed through either echocardiography or CT scan (as shown in Table 3), atrial dimensions exhibited no significant differences between the groups.

**Table 2:** Study sample comorbidities and group comparison.

Variable	Overall sample (*n* = 285)	Single puncture (*n* = 115)	Double puncture (*n* = 170)	*p*-value
Hypertension, *n* (%)	166 (58.2)	74 (64.3)	92 (54.1)	0.086
Hyperlipidemia, *n* (%)	95 (33.3)	46 (40)	49 (28.8)	0.050
Diabetes mellitus, *n* (%)	20 (7)	7 (6.1)	13 (7.6)	0.613
Obstructive sleep Apnea, *n* (%)	38 (13.3)	12 (10.4)	26 (15.3)	0.236
Smoking, *n* (%)	31 (10.9)	17 (14.8)	14 (8.2)	0.082
COPD, *n* (%)	10 (3.5)	6 (5.2)	4 (2.4)	0.210
Thyroid disease, *n* (%)	32 (11.3)	15 (13)	17 (10.1)	0.445
Cancer, *n* (%)	14 (4.9)	4 (3.5)	10 (5.9)	0.357
LVEF < 35%, *n* (%)	11 (4.1)	7 (6.4)	4 (2.5)	0.128
LVEF < 50%, *n* (%)	36 (13.4)	18 (16.5)	18 (11.3)	0.221
Heart failure, *n* (%)	22 (7.7)	11 (9.6)	11 (6.5)	0.337
Coronary disease, *n* (%)	19 (6.7)	10 (8.7)	9 (5.3)	0.264
Vascular disease (stroke, PAD, aortic plaques), *n* (%)	21 (7.4)	11 (9.6)	10 (5.9)	0.243
Previous stroke/TIA, *n* (%)	13 (4.6)	9 (7.9)	4 (2.4)	0.028
Hypertrophic cardiomyopathy, *n* (%)	7 (2.5)	3 (2.6)	4 (2.4)	0.589

COPD, chronic obstructive pulmonary disease; LVEF, left ventricular ejection fraction; PAD, peripheral arterial disease; TIA, transient ischemic attack.

**Table 3:** Echocardiography and computed tomography characteristics of the left atrium and group comparison.

Parameter	Overall sample (*n* = 285)	Single puncture (*n* = 115)	Double puncture (*n* = 170)	*p*-value
ECO LA diameter (mm)—mean ± SD	44 ± 8.5	44.8 ± 8.4	44.5 ± 6.8	0.819
ECO LA indexed volume ml/m^2^—mean ± SD	39.5 ± 17.6	39.1 ± 12.6	39.4 ± 16.5	0.943
CT scan LA volume in ml—mean ± SD	115.9 ± 42.5	106.5 ± 33.3	101.2 ± 33	0.245

ECO, echocardiography; LA, left atrium; CT, computed tomography.

Regarding the overall procedure duration (as presented in Table 4), the double TSP group had a slightly shorter duration (*p* = 0.008). However, the single TSP group substantially reduced fluoroscopy time and dwell time (*p* < 0.001).

**Table 4:** Group comparison of procedure characteristics regarding time and complications.

Parameter	Overall sample (*n* = 285)	Single puncture (*n* = 115)	Double puncture (*n* = 170)	*p*-value
Procedure duration in minutes—mean ± SD	131 ± 33.9	133 ± 31.7	123 ± 35.5	0.008
Fluoroscopy time in minutes—mean ± SD	16 ± 8.4	13 ± 6.3	19 ± 9.1	<0.001
Dwell time in minutes—mean ± SD	118 ± 95.4	113.9 ± 32.5	120.2 ± 108.0	<0.001
Major complications rate—*n* (%)	7 (2.4)	3 (2.6)	4 (2.3)	0.799

In the single TSP group, 90% of the cases underwent a second endocardial map after the first pass point-by-point ablation, and 29% received a third map due to gaps. Regarding the number of points collected during mapping with the high-resolution catheter, the average number of points was: 1,020 (388–2,200) for the first map, 1,009 (244–2,617) for the second map and 399 (135–1,019) for the third map (including only PV with gaps).

### 4.2. Complications occurrence

When we examine the occurrence of complications, major complications show similar rates in both groups (2.6% vs. 2.3%, with a *p*-value of 0.799). Procedural and periprocedural minor complications were observed in 12 patients (2.6% vs. 5.2%, with a *p*-value of 0.154). Minor complications included 6 cases (2.1%) of minor hemorrhage at the access site, 2 cases (0.7%) of pseudoaneurysm formation, 1 case (0.3%) of temporary right phrenic palsy, 1 case (0.3%) of air embolism in the coronaries, 1 case (0.3%) of hemoptysis, and 1 case (0.3%) of transient asystole during the procedure (refer to Table 5).

**Table 5:** Complication rates disaggregated by type of transeptal puncture.

Complication (*n* = 12)	Single puncture (*n* = 3)	Double puncture (*n* = 9)
Minor haemorrhage access site	2	4
Pseudoaneurysm	1	1
Temporary right phrenic palsy	0	1
Air embolism to the coronaries	0	1
Haemoptysis	0	1
Transitory asystole	0	1

### 4.3. Follow-up

**Figure 3:**
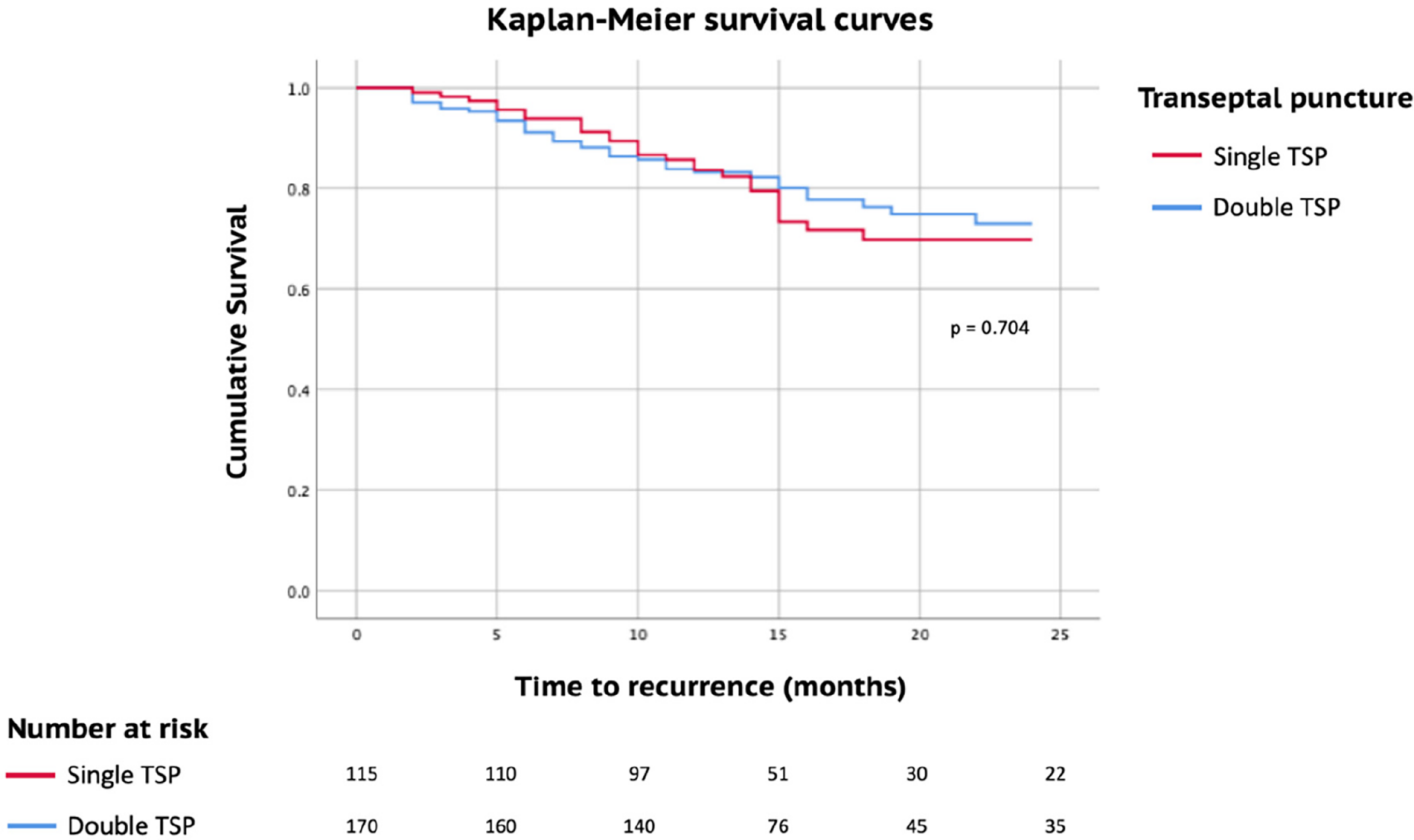
Kaplan meier survival curves estimates of AF recurrence during a mean follow-up of 24 months stratified by transeptal puncture: single vs. double (log-rank, *p* = 0.704).

The median follow-up period lasted 24 months, with a range from 12 to 48 months. At the 2-year follow-up mark, both groups displayed a similar rate of maintaining sinus rhythm (76.5% compared to 78.8%, with a *p*-value of 0.646). The assessments of atrial fibrillation recurrence over a 24-month follow-up in AF patients who underwent catheter ablation, stratified by transeptal puncture (single vs. double), revealed no distinction between the two approaches (*p* = 0.704) (Figure 3). Also, when examining the recurrence rates stratified by transeptal puncture (single vs. double) and the type of AF (paroxysmal vs. persistent), we observed no noteworthy differences between the groups (log-rank test, *p* = 0.113).

## 5. Discussion

### 5.1. Safety and feasibility of the single transeptal puncture approach in AF ablation

The present study demonstrated the feasibility and safety of a simplified single TSP puncture using 3D high-density PVI mapping using a point-by-point RF approach in a large cohort of AF patients. In addition, we compared double-TSP catheterisation, used in many centres as a standard technique, with a single TSP technique, using a 3D high-density mapping catheter and the ablation catheter through a single sheath (each catheter at a time).

There was not a substantial distinction between the two approaches in terms of the overall procedure duration, although the single puncture strategy did result in a slightly lengthier procedure time (133 ± 31.7 compared to 123 ± 35.5, with a *p*-value of 0.008). However, the extent of this difference (10 min) may not hold clinical significance. We attribute this variance to the operators’ initial learning curve, as it might be necessary to change catheters multiple times in the single puncture strategy, leading to a marginal increase in the total procedure time. Nevertheless, there was a notable reduction in dwell time in the single puncture group (*p* < 0.001).

It is crucial to emphasize that fluoroscopy times were significantly lower in the single transeptal puncture strategy. This substantial reduction in fluoroscopy time is important for patients and medical operators, as this decreased radiation exposure is linked to reduced risks (26).

The transeptal puncture (TSP) is a pivotal step in the atrial fibrillation (AF) ablation procedure. In theory, the double catheterization technique could introduce additional risks. The documented occurrence of pericardial effusion during catheter-based AF ablation varies between 0.5% and 4%, with cardiac tamponade observed in approximately 1% of cases (27, 28). A recently published meta-analysis of randomized clinical trials (29), which included patients undergoing their initial atrial fibrillation ablation procedure, revealed an overall complication rate of 4.5%. In this analysis, the incidence of severe procedure-related complications was 2.4%, which aligns with our findings.

Despite the need to exchange catheters a few times during the procedure, no stroke cases occurred in any group. Ischemic stroke complications can have dramatic consequences in AF ablation, with an incidence reported to be between 1% and 2% (30).

It’s important to emphasize that the authors did not employ adjunctive tools for transeptal puncture (TSP). The use of intracardiac echocardiography and transesophageal echocardiography guidance could play a crucial role in reducing adverse events associated with TSP and facilitating challenging transseptal catheterization (31). These imaging tools theoretically could have prevented some of the adverse events observed in our study. Further investigations with larger sample sizes may provide more clarity on this matter.

The single TSP approach resulted in fewer minor complications than the double-TSP catheterisation patients (2.6% vs. 5.2%), although this difference did not reach statistical significance. However, apart from air embolisation, it’s challenging to establish a clear correlation between the technique used to access the atrium and the occurrence of other complications. The absence of air embolisation complications in the single puncture group is noteworthy. It can be attributed to meticulous sheath irrigation throughout the procedure and careful aspiration during catheter changes.

Another aspect worth highlighting is that our centre did not have access to steerable sheaths that could be visualised using an electroanatomical mapping system when these procedures were conducted. Evidence indicates that this sheath type can substantially reduce fluoroscopy time (32).

### 5.2. Long-term outcome after AF catheter ablation

PVI, the cornerstone of any AF ablation procedure for paroxysmal and persistent AF, was the strategy therapy followed in this cohort. The AF/AT recurrence rate 24 months after the ablation was less than 30% for the entire cohort. In the current study, the AF/AT-free survival rate was slightly improved compared to other studies with long-term follow-up available in the literature. Kardos et al., in a study (33) comparing RF energy using contact force sensing and cryoballoon ablation for PVI in paroxysmal AF patients, showed freedom from AF/AT after a 24-month follow-up of around 66%. Ganesan et al.’s systematic review and meta-analysis included 6,167 patients with paroxysmal and non-paroxysmal AF from 19 studies undergoing AF ablation, with a mean follow-up of ≥24 months. They found freedom from AF/AT of 53% after one CA, with lower success rates for non-paroxysmal AF (3).

It should also be noted that both techniques have similar recurrence rates at 24 months follow-up. This comparable long-term outcome suggests that the single TSP technique is not sacrificing effectiveness for the sake of its other advantages. This is crucial for the overall success and sustainability of the ablation procedure.

Both groups equally distributed the operator’s experience, with ≥5 years of AF ablation procedures. This equal distribution minimises the potential bias related to operator experience, making the comparison more reliable.

We would also like to point out that a steerable sheath was used in the single TSP group. In contrast to a non-steerable sheath, the use of steerable sheath technology enhances control over catheter manipulation. This advancement theoretically enables a broader spectrum of catheter orientations and improved stability, potentially leading to enhanced tissue contact and, consequently, more effective ablation lesions. One systematic review and meta-analysis that analysed the results of using both strategies found that the fluoroscopic and RF application times were comparable (34). Another recent meta-analysis (35) also considered comparing these two technologies for RF ablation of AF. It concluded that steerable sheaths can effectively reduce the recurrence rate of AF and the occurrence of acute PVs reconnection events. However, there is no advantage in shortening the total RF time, fluoroscopy time, total surgical time and reducing complications (35). Considering that our study aimed to evaluate the complications and long-term results, using the steerable sheath might influence the recurrence rate but not the other parameters reported in our results.

### 5.3. Study limitations

We wish to emphasise several limitations in our research. Firstly, this study was conducted as a non-randomized single-centre investigation. However, it's worth noting that both groups consisted of atrial fibrillation patients who were consecutively enrolled for point-by-point pulmonary vein isolation, which somewhat mitigates the absence of randomisation. Secondly, the significant advancements in 3D mapping systems and the increasing experience of the operators over the course of the study, which spanned five years, may have influenced the observed reduction in radiation exposure times. It's important to highlight that our primary objective was to assess the safety of catheter ablation, specifically focusing on single-transseptal puncture (TSP) and double-TSP approaches. Thirdly, the utilisation of 24 h Holter recordings, as opposed to 7-day recordings, could potentially lead to an underestimation of AF recurrence rates. Nevertheless, it is noteworthy that this assessment method has been employed by multiple researchers, and in our study, it was consistently applied to both groups, minimising the likelihood of introducing substantial bias. Fourthly, it is important to point out that a steerable sheath was used in the single TSP group, which may introduce some variance in the comparison between the groups.

Fifthly we did not perform pre- and post-catheter ablation cerebral magnetic resonance imaging in this study. Consequently, we could not evaluate the incidence of silent cerebral lesions (36), which could be significant, especially in catheter exchange within the single transeptal puncture group. Lastly, while our approach theoretically simplifies the catheter manipulation process, it is important to acknowledge that it may demand a longer learning curve than conventional techniques, which should be considered when implementing this method.

## 6. Conclusion

This retrospective analysis of a large cohort of consecutive patients highlights the viability, safety, and high success rate of employing a single transeptal puncture and a single sheath for catheter placement in the left atrium during atrial fibrillation ablation. This approach represents a valuable alternative to the conventional practice of using double transseptal catheterisation and double sheaths simultaneously. Moreover, it offers an option for operators seeking to reduce x-ray exposure and minimise potential complications associated with a second TSP procedure while delivering promising long-term results for paroxysmal and persistent AF.

In summary, the single TSP technique presents advantages such as reduced fluoroscopy time, the potential avoidance of acute complications, and comparable long-term outcomes. These findings indicate that the single TSP approach is feasible and preferable in certain aspects, contributing to the enhancement and optimisation of AF ablation procedures.

## Data Availability

The raw data supporting the conclusions of this article will be made available by the authors, without undue reservation.
